# Dysrupted microbial tryptophan metabolism associates with SARS-CoV-2 acute inflammatory responses and long COVID

**DOI:** 10.1080/19490976.2024.2429754

**Published:** 2024-11-17

**Authors:** Lu Yao, Hannah Devotta, Junhui Li, Nonhlanhla Lunjani, Corinna Sadlier, Aonghus Lavelle, Werner C. Albrich, Jens Walter, Paul W. O’Toole, Liam O’Mahony

**Affiliations:** a School of Microbiology, University College Cork, Cork, Ireland; b APC Microbiome Ireland, University College Cork, Cork, Ireland; c Department of Dermatology, University Hospital Limerick, Limerick, Ireland; d Department of Medicine, University College Cork, Cork, Ireland; e Department of Infectious Diseases, Cork University Hospital, Cork, Ireland; f Department of Anatomy and Neuroscience, University College Cork, Cork, Ireland; g Division of Infectious Diseases & Hospital Epidemiology, Cantonal Hospital St. Gallen, St. Gallen, Switzerland

**Keywords:** Microbiota, COVID-19, tryptophan, indoles, long COVID

## Abstract

Protection against severe acute respiratory syndrome coronavirus 2 (SARS-CoV-2) infection and risk of long COVID has been associated with the depletion or over-abundance of specific taxa within the gut microbiome. However, the microbial mechanisms mediating these effects are not yet known. We hypothesized that altered microbial production of tryptophan and its downstream derivatives might contribute to inappropriate immune responses to viral infection. In patients hospitalized with COVID-19 (*n* = 172), serum levels of tryptophan and indole-3-propionate (IPA) negatively correlated with serum levels of many proinflammatory mediators (including C-reactive protein and Serum amyloid A), while C-glycosyltryptophan (C-Trp), indole-3-lactic acid (ILA) and indole-3-acetic acid (IAA) levels were positively correlated with levels of acute phase proteins, proinflammatory cytokines, alarmins and chemokines. A similar pattern was observed in long COVID patients (*n* = 20) where tryptophan and IPA were negatively associated with a large number of serum cytokines, while C-Trp and IAA were positively associated with circulating cytokine levels. Metagenomic analysis of the fecal microbiota showed the relative abundance of genes encoding the microbial enzymes required for tryptophan production (e.g. anthranilate synthase) and microbial tryptophan metabolism was significantly lower in patients hospitalized with COVID-19 (*n* = 380) compared to healthy controls (*n* = 270). Microbial tryptophan metabolites reduced innate cell proinflammatory responses to cytosolic DNA sensor Stimulator of interferon genes (STING), toll-like receptor (TLR)-3 and TLR-4 stimulation *in vitro*, while IL-10 secretion was enhanced. Microbial tryptophan metabolites also modified *ex vivo* human lymphocyte responses by limiting the production of TH1 and TH17 associated cytokines, while enhancing secretion of IL-22. These data suggest that lower levels of tryptophan production and tryptophan metabolism by gut microbes may increase the risk of severe and chronic outcomes to SARS-CoV-2 infection due to impaired innate and adaptive responses to infection. Screening patients for lower-level microbiome capacity for tryptophan metabolism may help identify at-risk individuals.

## Introduction

Evolution of mammals with symbiotic microbial communities has resulted in intertwined metabolic pathways that affect physiological and pathological processes in the host. Over 60% of human blood metabolites are significantly associated with either host genetics or the gut microbiome, with an estimated 69% of these associations driven solely by the microbiome.^1^ Microbial fermentation of dietary components *in vivo* generates thousands of molecules, some of which are integral components of the molecular circuitry that regulates immune and metabolic functions.^2,3^ For example, microbiota-derived metabolites such as taurine, histamine, and spermine were shown to modulate NLR Family Pyrin Domain Containing 6 (NLRP6) inflammasome signaling, epithelial IL-18 secretion, mucosal IL-4 responses, and eosinophil activation.^4–6^ Treatment of human dendritic cells with the bacterial-derived lipid 12,13-dihydroxy-9Z-octadecenoic acid (12,13-diHOME – an oxylipin terminal product of linoleic acid metabolism) reduced anti-inflammatory cytokine secretion and the number of Treg cells, suggesting that this metabolite impedes immune tolerance.^7^ In contrast, short chain fatty acids (SCFAs) promote IL-10 secretion by dendritic cells, increase regulatory T cell (Treg) numbers and effectiveness, influence bone marrow hematopoiesis, reduce effector T cell activity, improve epithelial barrier, and inhibit mast cell and type 2 innate lymphoid cells (ILC2) activation.^8–12^ SCFAs exert their effects by binding to G protein-coupled receptors (GPCRs) such as GPR41, GPR43 and GPR109A, and through epigenetic modifications. Tryptophan derivatives of diet, host and microbial origin are emblematic of this metabolic crosstalk.^13–15^ Tryptophan is an essential amino acid that must be supplied in the diet and can be generated at low levels by microbes within the gut.^16–18^ Tryptophan is metabolized through two primary host pathways, the serotonin (5-HT) pathway and the kynurenine pathway, while microbes can generate a wide range of indole derivatives following tryptophan metabolism.^19^ Some microbial-derived tryptophan metabolites (e.g. indole-3-propionic acid (IPA) and indole-3-aldehyde (IAAld)) can trigger host aryl hydrocarbon receptor (AhR) responses resulting in cytokine secretion that improves the epithelial barrier and limits local inflammatory responses including TH17 CD4 T cell differentiation.^20–22^ Due to the complexity of the human microbiome and its vast coding potential, these examples provide the rationale for the systematic identification of additional bacterially produced molecules that mediate microbiota effects on the host immune system. Indeed, one systematic analysis identified over 3,000 small molecule biosynthetic gene clusters within the human microbiome.^23^ Remarkably, the majority of these gene clusters have not been further studied, and even for the metabolites described above, mechanistic and causal information on their effects in humans is limited.

Infection with viruses such as SARS-CoV-2 leads to a wide variety of potential outcomes from asymptomatic responses to acute respiratory distress and death.^24,25^ However, several risk factors for severe COVID-19 have been identified (e.g. age, obesity), the pathophysiological mechanisms that contribute to disease severity are not fully understood, although two leading hypotheses have emerged.^26^ First, an inability to mount an effective immune response in a timely manner seems important. Second, an inability to control SARS-CoV-2-driven inflammatory responses results in excessive levels of inflammatory molecules that damage the vasculature, limit organ function and restrict homeostatic and repair mechanisms. In addition, we now appreciate that the long-term consequences of viral infection (long COVID) can affect multiple organ systems for years after the initial virus infection.^27–30^

We and others have previously shown that the composition and metabolic activity of the gut microbiota may be closely related to the severity of acute COVID-19 and the risk of long-term consequences.^31–36^ These studies support the concept that successful responses to infectious agents such as SARS-CoV-2 involve the gut microbiome and might be mediated by the effect of microbial-derived metabolites on innate and adaptive immune responses. In this study, we hypothesized that microbial production of tryptophan and its downstream metabolites might restrain the devastating overproduction of inflammatory cytokines and soluble mediators, which leads to multiorgan failure or long COVID. We reanalyzed cytokine and metabolomic data we previously published for hospitalized COVID-19 patients, long COVID patients and healthy volunteers, specifically focusing on microbial tryptophan metabolites.^31,35^ In addition, we examined microbial tryptophan metabolism using data from 8 shotgun metagenomic studies that were previously included in our meta-analysis of gut microbiome associations with COVID-19 severity.^36^ We show significant associations between tryptophan and its derivatives with circulating immune mediators, while lower abundance of microbial genes required for tryptophan biosynthesis and metabolism is evident in hospitalized COVID-19 patients. Lastly, we show using *in vitro* models that these microbial tryptophan metabolites modulate inflammatory responses relevant to virus infection.

## Results

### Altered serum levels of microbial tryptophan metabolites in COVID-19 and long COVID patients

While profound differences in circulating metabolites and cytokines due to SARS-CoV-2 infection are already well described, we focused on associations between immune activation and microbial tryptophan metabolites. Serum levels of IPA and IAA were lower in hospitalized patients with SARS-CoV-2 infection (*n* = 172) compared to healthy controls (*n* = 29) and levels were lowest in those with severe disease, while ILA levels were lower in COVID-19 patients but not in those with a fatal outcome to infection (Fig. S1). Serum tryptophan levels primarily reflect the combined outcomes of dietary intake and utilization by host metabolism, and tryptophan levels were negatively associated with COVID-19 severity. C-Trp results from host carbon directed glycosylation of tryptophan, and C-Trp levels positively correlated with severity (Fig. S2). Microbe-derived indole is metabolized by the liver into 5-hydroxyindole sulfate (H5S) and 3-indoxyindole sulfate (I3S), which were both present at lower levels in COVID-19 patients, but their levels did not correlate with disease severity (Fig. S3). Differences in microbial tryptophan metabolite levels remained evident in long COVID patients (*n* = 20 at two timepoints) where tryptophan and IPA levels were consistently lower than those of controls (*n* = 20), while C-Trp and IAA levels were significantly higher than controls (Fig. S4). The long COVID patients included in this study were selected according to the WHO criteria – the continuation or development of new symptoms 3 months after the initial SARS-CoV-2 infection, with these symptoms lasting for at least 2 months with no other explanation.

### Microbial tryptophan metabolites correlate with serum cytokines

**Figure 1.**
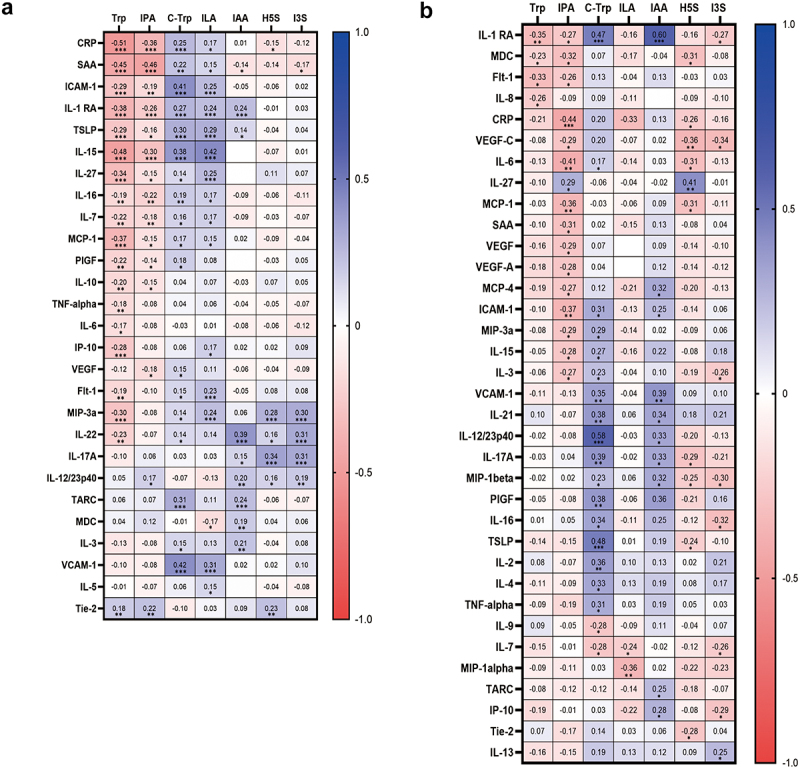
Tryptophan metabolites correlate with cytokine levels.

In order to determine if microbial tryptophan metabolism might associate with specific patterns of immune activation during SARS-CoV-2 infection, we performed a correlation analysis of metabolites versus serum cytokines (*n* = 172 COVID-19 patients and *n* = 29 controls). We have previously shown that many circulating cytokines increase with COVID-19 severity (Fig. S5). Circulating levels of tryptophan and IPA negatively correlated with many proinflammatory mediators (including CRP and SAA), thymic stromal lymphopoietin (TSLP) alarmin levels and cell migration factors (e.g. soluble Intercellular Adhesion Molecule (ICAM)-1 and monocyte chemoattractant protein (MCP)-1) (Figure 1a). In contrast, circulating levels of C-Trp, ILA and IAA were positively correlated with acute phase proteins, proinflammatory cytokines, alarmins and chemokines (Figure 1a). H5S and I3S negatively correlated with acute phase protein levels and positively correlated with immune mediators associated with TH17 responses. A similar pattern was observed in long COVID patients (*n* = 20 patients and *n* = 20 controls) where tryptophan and IPA were negatively associated with a large number of serum cytokines, while C-Trp and IAA were positively associated with circulating cytokine levels (Figure 1b). ILA, H5S and I3S were negatively associated with specific cytokines (Figure 1b).

Serum tryptophan metabolites and serum cytokine levels correlate in acute COVID-19 patients (a) and long COVID patients (b) using Pearson correlation calculations and a two-tailed *p* value with correction for multiple comparisons. Red indicates a negative correlation and blue indicates a positive correlation. Trp – Tryptophan; IPA – indole-3-propionate; C-Trp – C-glycosyltryptophan; ILA – indole-3-lactic acid; IAA – indole-3-acetic acid; H5S - 5-hydroxyindole sulfate; I3S - 3-indoxyl sulfate. *p < 0.05; **p < 0.01; ***p < 0.001

### Identification of genes associated with tryptophan metabolism within the gut microbiome

To investigate the association between relative abundance of genes specifically encoding tryptophan associated enzymes with COVID-19, we examined 8 shotgun metagenomic studies that were previously included in our meta-analysis of gut microbiome associations with COVID-19 severity (Supplementary Table S1).^36^ The available patient metadata is summarized in Table 1. Age, body mass index (BMI) and sex were similar for controls and COVID-19 patients, while COVID-19 patients with severe disease were significantly older and more often male. Antibiotic use was more frequent in COVID-19 patients compared to controls. We only included the first sampling timepoint following hospitalization for each patient. To ensure consistency in the bioinformatic analyses, all metagenomic sequencing data were reprocessed and functionally profiled for gene families and pathways using HUMAnN3. Genes involved in tryptophan metabolism were identified using MetaCyc^37^ and the genes identified for analysis are listed in supplementary Table S2.

**Table 1.** Participant metadata for metagenomic study analysis.

			COVID Severity		
	Control *n* = 270	COVID *n* = 380	Mild/Moderate *n* = 217	Severe/Fatal *n* = 49	Uncategorized Severity *n* = 114
Age Median (SD)	47.0 (13.2) *n* = 133	40.0 (19.2) *n* = 237	38.5 (17.8) *n* = 200	62.0 (15.2)* *n* = 37	Not Recorded
BMI Median (SD)	21.3 (2.4) *n* = 32	22.8 (2.7) *n* = 59	22.4 (2.8) *n* = 48	23.4 (2.8) *n* = 11	Not Recorded
Male:Female Ratio	1:0.96 *n* = 133	1:0.81 *n* = 237	1:0.90 *n* = 200	1:0.43* *n* = 37	Not Recorded
Antibiotics Yes/No	0/118	94:159*	59:148*	36:11*	Not Recorded
Diabetes Yes/No	0/8	7/56	5/45	2/11	Not Recorded
Hypertension Yes/No	0/8	18/45	13/37	5/8*	Not Recorded

*p < .05 compared to controls. SD – standard deviation.The number of participants with metadata available is indicated by the “n” value.

**Figure 2.**
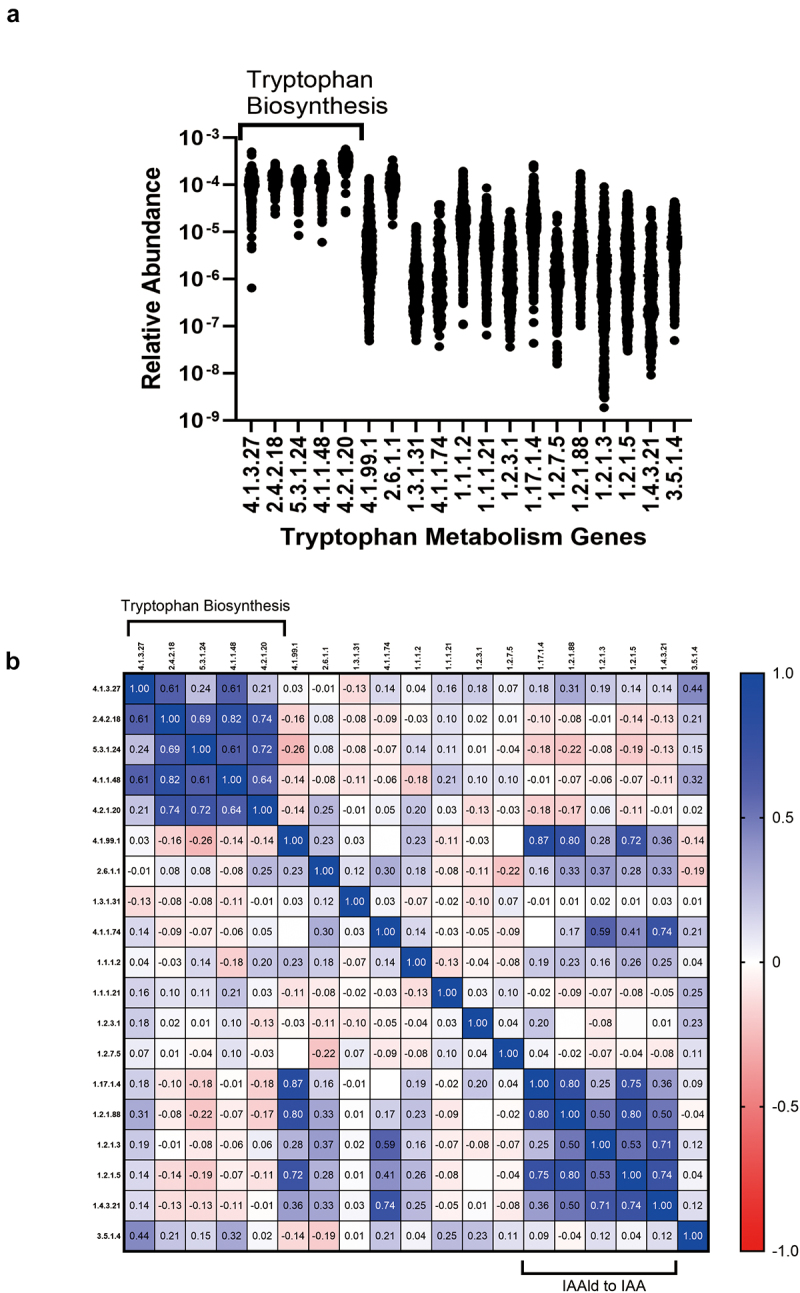
Microbial tryptophan metabolism genes.

Microbes can generate tryptophan from plant and microbial metabolic intermediates such as chorismate, and they can also metabolize tryptophan into a wide range of indole compounds. In total, 19 genes associated with tryptophan biosynthesis (5 genes) and metabolism (14 genes) were detected in greater than 10% of individuals whose microbiomes were analyzed (*n* = 650). The proportion of individuals that had detectable tryptophan biosynthesis or metabolism genes was different for each gene and ranged from 100% to 14% (Supplementary Table S3). Interestingly, the relative abundance of the five genes required for tryptophan biosynthesis was higher than those involved in tryptophan metabolism (Figure 2a). In addition, the relative abundance of all five tryptophan biosynthesis genes was highly correlated with each other, which may suggest all five genes were usually located in a single operon or were perhaps restricted to closely related microbes (Figure 2b). The relative abundance levels of a second group of five genes involved in IAAld to IAA metabolism were also closely correlated with each other (Figure 2b). The relative abundance of the 19 genes was not associated with age, BMI or antibiotic use (supplementary Table S4). The relative abundance of 1 gene (1.1.1.21 – Aldose reductase) was significantly higher in females compared to males, while the relative abundance of the remaining 18 genes were not influenced by sex (Supplementary Table S4).

The relative abundance of 19 genes associated with tryptophan metabolism in healthy volunteers and COVID-19 patients (*n* = 650) are shown (a) E.C. numbers are included on the x-axis. (b) Pearson correlation matrix showing two clusters of genes whose relative abundance correlates with each other. Numbers illustrated are Pearson correlation coefficients. IAA – indole-3-acetic acid; IAAld – Indole-3-aldehyde.

### Microbial tryptophan biosynthesis genes relative abundance is reduced in COVID-19 patients

**Figure 3.**
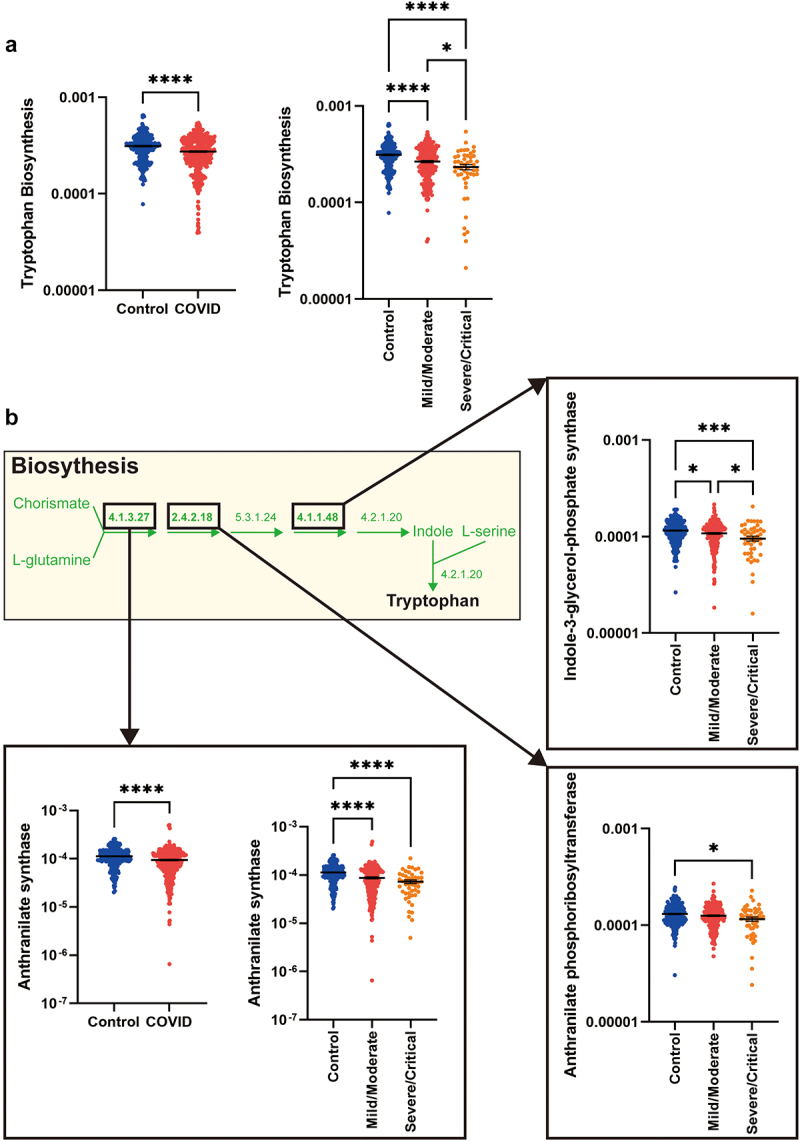
Microbial genes for tryptophan biosynthesis.

We performed pathway analysis focused on tryptophan biosynthesis and demonstrated significantly reduced pathway gene occupancy and pathway completeness in patients with COVID-19 (*n* = 380) compared to healthy controls (*n* = 270) and in patients with severe COVID-19 compared to those with mild/moderate disease (Figure 3a). This difference was not driven by study-specific factors, as reduced tryptophan biosynthesis was observed in each study when also analyzed individually (Fig. S6). In addition to performing pathway analysis, we assessed gene counts for each of the pathway steps. Of the five genes required for the synthesis of tryptophan from chorismate, the relative abundance of *trpD* (encoding anthranilate synthase) was significantly lower in patients with COVID-19 (*n* = 380) compared to healthy controls (*n* = 270) (Figure 3b). Levels were lower in hospitalized patients with mild/moderate disease (*n* = 217) and those with severe/fatal COVID-19 (*n* = 49). Genes whose products act further downstream in the tryptophan biosynthesis pathway encoding for the enzyme anthranilate phosphoribosyltransferase and indole-3-glycerol-phosphate synthase were at significantly lower abundance in COVID-19 patients with severe disease compared to controls (Figure 3b).

(a) Relative abundance of all genes in the tryptophan biosynthesis cluster were significantly decreased in COVID-19 patients compared to controls, and in those with severe disease compared to those with mild/moderate disease. (b) The microbial tryptophan biosynthesis pathway is illustrated and E.C. numbers are included for each gene. The relative abundance of genes that are significantly different in COVID-19 patients, or in those with severe disease are highlighted and dot plots are included for those that are significantly different. Results are expressed as mean and standard deviation. Differences between groups were calculated using the Kruskal–Wallis test and Dunn’s multiple comparison test. *p < .05; ***p < .001; ****p < .0001

### Microbial tryptophan metabolism genes relative abundance is altered in COVID-19 patients

**Figure 4.**
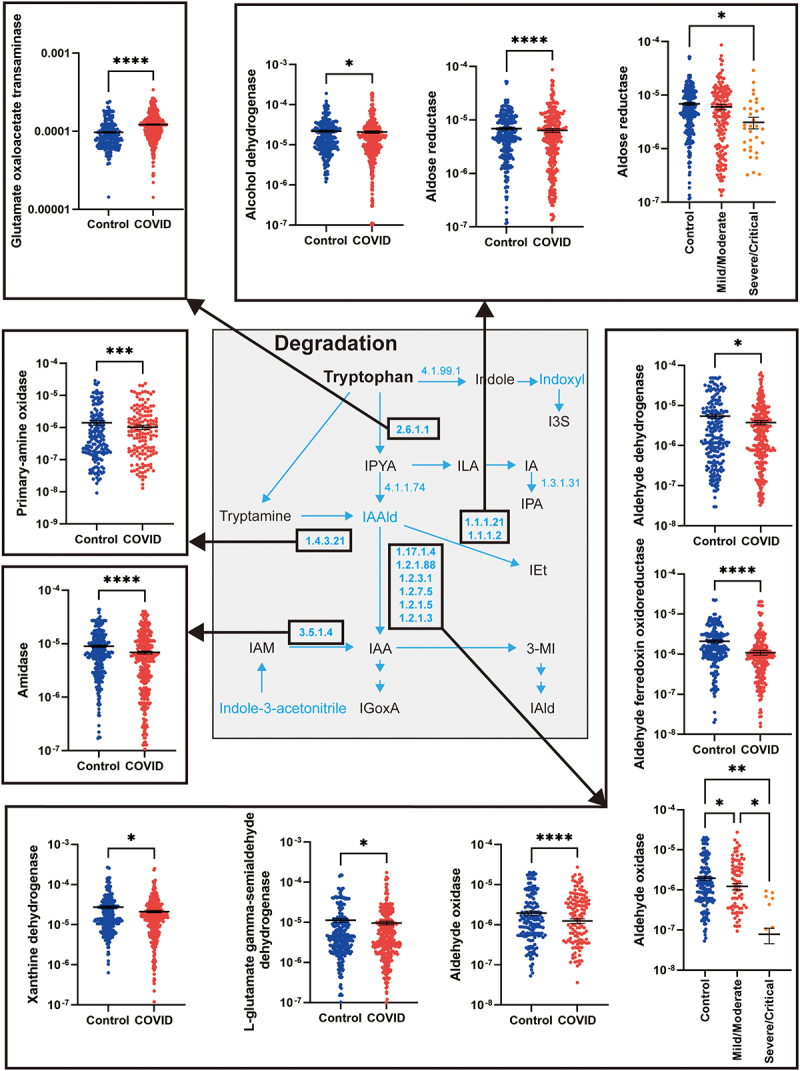
Microbial genes for tryptophan metabolism.

Fourteen genes involved in microbial metabolism of tryptophan into indoles were analyzed. Of these genes, the relative abundance of one gene was significantly higher in COVID-19 patients compared to controls, four genes were at similar levels for patients and controls, while the relative abundance of nine genes was significantly reduced in the gut microbiota of COVID-19 patients (Figure 4). The relative abundance of the *aspC* gene encoding for glutamate oxaloacetate transaminase, which is one of the enzymes required for metabolism of tryptophan into indole-3-pyruvic acid (IPYA), was significantly higher in COVID-19 patients. In contrast, genes encoding enzymes involved in the metabolism of IAAld to indole-3-ethanol (IEt), IAAld to IAA, indole-3-acetamide (IAM) to IAA, and tryptamine to IAAld were at a significantly lower relative abundance in COVID-19 patients (Figure 4). Importantly, the relative abundances of genes encoding for aldose reductase and aldehyde oxidase were significantly lower in COVID-19 patients with severe disease.

The KEGG microbial tryptophan metabolism pathways are illustrated, and E.C. numbers are included for each gene included in the analyses. The relative abundance of genes that are significantly different in COVID-19 patients, or in those with severe disease are highlighted by a black box, and associated dot plots are linked by an arrow for those genes that are significantly different. Results are expressed as mean and standard deviation. Differences between groups were calculated using the Kruskal–Wallis test and Dunn’s multiple comparison test. *p < .05; **p < .01; ***p < .001; ****p < .0001

### Microbial tryptophan metabolites directly influence innate anti-viral immune responses

In order to determine if changes in levels of microbial tryptophan metabolites could directly influence host immune responses relevant to infection, we performed *in vitro* cultures of stimulated immune cells with these tryptophan metabolites.

**Figure 5.**
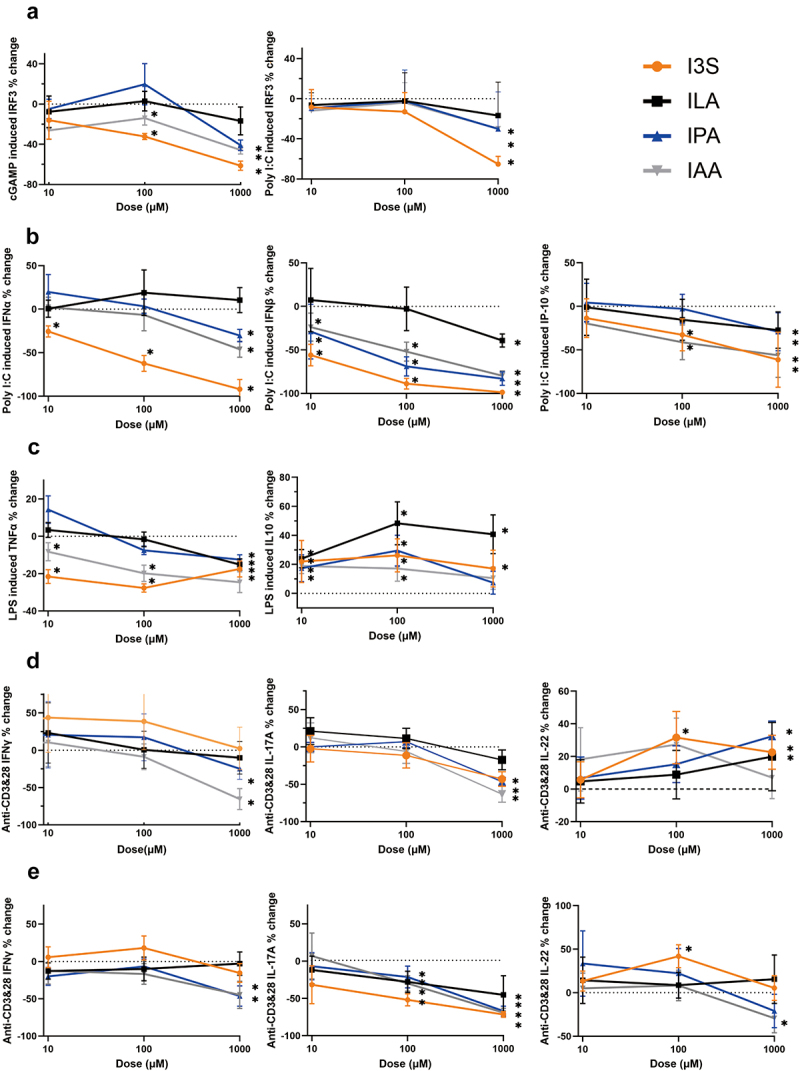
Microbial tryptophan metabolites influence immune cell responses.

Interferon regulatory factor 3 (IRF3) is an interferon regulatory transcription factor that plays an important role in coordinating the innate immune response to viral infection. Cyclic dinucleotide [G(2’,5’)pA(3’,5’)p] (2’3’-cGAMP) was used to activate the cytosolic DNA sensor Stimulator of interferon genes STING, which induces type I interferons via the TANK-binding kinase 1 (TBK1)/IRF3 pathway. I3S, IPA, IAA and ILA were co-incubated with THP-1 cells, and they did not significantly impact cell viability up 1000 µM (data not shown). I3S, IPA and IAA, but not ILA, significantly reduced 2’3’-cGAMP-induced IRF3 activation in THP-1 cells (Figure 5a). In addition, polyinosinic:polycytidylic acid (Poly I:C – structurally similar to double-stranded RNA) was also used to activate IRF3 via retinoic-acid-inducible protein 1 (RIG-I)/melanoma-differentiation-associated gene 5 (MDA5) stimulation in THP-1 cells. A similar pattern to 2’3’-cGAMP activation was observed whereby I3S was the most inhibitory, followed by IPA, IAA and ILA (Figure 5a). In contrast, nuclear factor kappa B (NF-kB) activation in THP-1 cells following lipopolysaccharide (LPS) stimulation of cell surface TLR-4 was not influenced by I3S, IPA, IAA or ILA (Fig. S7).

Metabolites were co-incubated with cells at 10, 100, or 1000 µM. (a) IRF3 activation in THP-1 cells (*n* = 4). (b) Poly I:C stimulated cytokine secretion by PBMCs (*n* = 4). (c) LPS stimulated cytokine secretion by PBMCs (*n* = 4). (d) Anti-CD3 and anti-CD28 stimulated cytokine secretion by PBMCs (*n* = 6). (e) Anti-CD3 and anti-CD28 stimulated cytokine secretion by CD4^+^ T cells (*n* = 6). Results are shown as % change from stimulated positive control and expressed as mean and standard deviation. IPA – Indole-3-propionate; ILA – Indole-3-lactic acid; IAA – Indole-3-acetic acid; I3S 3-indoxyl sulfate. *p < .05

Human peripheral blood mononuclear cells (PBMCs) were stimulated with Poly I:C or LPS and cytokine secretion measured (2’3’-cGAMP did not induce significant cytokine secretion from PBMCs). I3S was the most potent metabolite in reducing Poly I:C induced interferon (IFN)-α, IFN-β and interferon gamma-induced protein (IP)-10 secretion from PBMCs (Figure 5b). IAA and IPA also reduced Poly I:C stimulated cytokine secretion, but higher doses were required compared to I3S. ILA reduced IFN-β and IP-10 secretion only at 1000 µM and had no effect on IFN-α secretion. I3S and IAA significantly reduced LPS-stimulated PBMC secretion of the proinflammatory cytokine tumor necrosis factor (TNF)-α at 100 µM, while all four tested metabolites reduced TNF-α secretion at 1000 µm (Figure 5c). In contrast, I3S, IPA, IAA and ILA enhanced PBMC secretion of the regulatory cytokine IL-10, even at the lowest dose tested of 10 µM (Figure 5c).

### Microbial tryptophan metabolites directly influence lymphocyte polyclonal immune responses

Lastly, PBMCs (Figure 5d) or purified CD4^+^ lymphocytes (Figure 5e) were activated with anti-human CD3 and CD28 antibodies to stimulate lymphocyte cytokine secretion. IAA and IPA significantly reduced IFN-γ secretion, while I3S, IAA and IPA reduced IL-17A secretion at the highest dose used in stimulated PBMCs (Figure 5d). In contrast, I3S, IPA and ILA enhanced secretion of IL-22, which is a cytokine important for epithelial barrier function. Similar responses were observed for purified CD4^+^ lymphocytes, where IAA and IPA reduced IFN-γ secretion and all four metabolites reduced IL-17A secretion in a dose-dependent manner (Figure 5e). I3S enhanced secretion of IL-22 from CD4^+^ T cells, while IAA reduced IL-22 secretion (Figure 5e).

**Figure 6.**
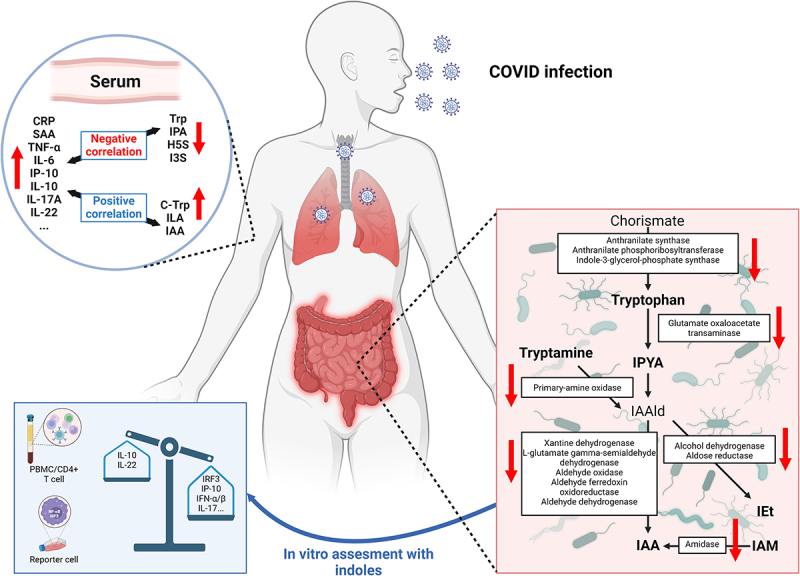
Circulating levels of microbial tryptophan metabolites correlated with inflammatory markers and COVID-19 severity. Similarly, the relative abundance of bacterial genes encoding tryptophan synthesis and metabolism enzymes were underepresented in hospitalised COVID-19 patients. Bacterial-derived indoles modified activated immune cell responses by enhancing secretion of IL-10 and IL-22, while reducing IRF3 mediated transcription and reduced secretion of IP-10, interferons and IL-17.

Figure 6 graphically illustrates the findings of this study.

## Discussion

In this study, we show that tryptophan and microbial generated tryptophan metabolites are associated with dysregulated immune responses to SARS-CoV-2 during hospitalization and these associations were still evident more than 6 months following infection in patients with long COVID. Reduced levels of genes that generate and metabolize tryptophan in COVID-19 patients may contribute to dysregulated tryptophan metabolism and altered levels of indoles. We also show that many of these metabolites influence innate and adaptive responses stimulated by viral infection, and altered levels *in vivo* may contribute to dysregulated inflammatory responses and associated tissue damage.

Tryptophan levels and metabolism are associated with optimal regulation of multiple physiological processes, including inflammation, metabolism, immune responses, and neurological function.^14,19^ Tryptophan serum levels are known to be influenced by dietary intake and activity of the three metabolic pathways (kynurenine, 5-hydroxytryptamine, and indole pathways). Indeed, increased activity of the interferon-induced indoleamine-2,3-dioxygenase (IDO) pathway in COVID-19 patients has been well described, and this pathway is likely responsible for significant depletion of tryptophan in patients with COVID-19.^38^ Reduced absorption of dietary tryptophan via the small intestine in murine models of viral infection was recently shown to contribute to decreased serum tryptophan levels.^39^ We found a high relative abundance of genes encoding for tryptophan biosynthesis (relative to tryptophan metabolizing genes) within the human gut microbiota. While microbial-generated tryptophan in the gut will not contribute substantially to host serum tryptophan levels, tryptophan generated by microbes will be available for microbial metabolism and potentially may also be available for host immune cell metabolism within the mucosa, although this is yet to be tested. Notably, antibiotic-driven depletion of the gut microbiome, and associated impacts on tryptophan metabolism, was shown to disrupt the induction of antibody responses to influenza vaccination possibly by driving inflammatory signaling in innate immune cells.^40^

In addition to tryptophan, IPA levels were inversely associated with many circulating cytokines in COVID-19 patients and long COVID patients, suggesting that IPA in particular may play an important role in modulating inflammatory responses to viral infection. IPA is exclusively generated by the gut microbiota so changes in gut microbial composition, as well as dietary patterns, can affect IPA production.^41,42^ IPA has already been suggested to play a role in a number of metabolic and immune mediated disorders in humans.^43^ A recent murine study showed that IPA levels inversely correlate with influenza viral load, and inflammation markers, while IPA supplementation in diseased animals reduced viral load and lowered local and systemic inflammation.^44^ While many of the tryptophan metabolites can activate the AhR, IPA can also signal through the pregnane X receptor (PXR).^45^ Our *in vitro* assays showed that IPA significantly modulates the activity of innate viral sensors and polyclonal T cell responses, including reducing IFN-α and IFN-β secretion. On the one hand, type 1 interferon responses are important for protection against virus infection – indeed SARS-CoV-2 antagonizes type 1 interferon responses as an immune evasion strategy.^46^ In contrast, type 1 interferon responses can also have harmful effects by instigating inflammatory responses or inducing T cell suppression during virus infections.^47^ However, the optimal timing and level of type 1 interferon secretion is currently unknown. Perhaps enhancing secretion of additional cytokines such as IL-10 and IL-22 that promote immune regulation and barrier function, in conjunction with partial suppression of type 1 interferons, may be crucial for protection against viral-induced tissue damage. Future studies are needed to further dissect the signaling mechanisms underpinning IPA effects on viral-induced inflammation and to determine if IPA will be beneficial early or later during the course of infection.

Recent industrialized western lifestyle changes, particularly dietary habits, have negatively affected microbiome communities and their metabolic output.^48–50^ Overall, species diversity and richness have been shown to be reduced by about one-third in Americans compared to Malawians or Amerindians.^51^ Adults who more regularly consumed plant-based or pescatarian diets seemed to have lower likelihood of developing severe COVID-19.^52–54^ However, the specific plant-based substrates (e.g. fiber type, fatty acids, and polyphenols) that are responsible for these positive associations are unknown. Microbial biosynthesis of tryptophan depends on the availability of chorismate, which is generated via the shikimate pathway.^55^ This pathway is restricted to bacteria, fungi, and plants, and is particularly important in vascular plants where up to 30% of photosynthetically fixed carbon is directed through this pathway.^56^ Thus, the shikimate pathway, chorismate, and tryptophan may represent an important nexus where diet, gut microbiota, and immune health intersect. It will be interesting to assess in future studies if chorismate intake from plant-based sources correlates with COVID-19 severity.

There are several limitations that should be noted. A relatively small number of long COVID patients were included in this study, which limits the generalizability of our findings. In particular, the long COVID population described here had been hospitalized during their acute disease and many long COVID patients develop disease after a mild course of SARS CoV2 infection. We do not yet know if our findings are also relevant for long COVID patients that were not hospitalized during acute infection. The correlation analyses between microbial metabolites and cytokine levels are associative and not necessarily indicative of causation. Multiple confounding factors may also influence our results, which include diet, medications, comorbidities and lifestyle – it is possible that these factors influence microbial tryptophan metabolism prior to SARS-CoV-2 infection.

Infection with SARS-CoV-2 leads to a wide variety of potential outcomes from asymptomatic responses to acute respiratory distress and death. This study supports the concept that the lack of tolerance promoting and effector molecules, such as microbial tryptophan metabolism, results in inadequate immune training and a hypersensitive immune system that responds inappropriately to challenges, such as infection with SARS-CoV-2. These immune regulatory metabolites are expected and evolutionarily hardwired into immune system decision-making processes. Future clinical studies should focus on promoting the production of microbial-derived tryptophan and indoles that promote early anti-viral defense responses and support the negative feedback mechanisms that restrain the devastating overproduction of inflammatory cytokines and soluble mediators, which lead to multiorgan failure. In particular, supplementation with tryptophan producing microbes in combination with appropriate dietary supports (e.g. chorismate) may represent a novel targeted approach for the prevention of aberrant inflammatory responses to acute infection and promote resolution of inflammation in patients with chronic symptoms such as those with long COVID.

## Materials and methods

### Metabolomic and cytokine correlations

We reanalyzed cytokine and metabolomic data we previously published for hospitalized COVID-19 patients (*n* = 174), long COVID patients (*n* = 20) and healthy volunteers (*n* = 29), specifically focusing on microbial tryptophan metabolites.^31,35^ Serum samples were collected immediately following enrollment, which was usually within 24–48 h of hospital admission. Briefly, untargeted metabolomics was performed on patient sera by Metabolon using the HD4 platform. Metabolite peaks were quantified for each sample using area-under-the-curve. Data was normalized for each metabolite by dividing the raw peak values in the experimental samples by the median value for all samples tested and the results are presented as median-scaled data. We also examined the levels of 54 cytokines and growth factors (using MSD multiplex kits according to manufacturer’s instructions) in serum, which included IL-1α, IL-1β, IL-1RA, IL-2, IL-3, IL-4, IL-5, IL-6, IL-7, IL-8, IL-9, IL-10, IL-12/23p40, IL-12p70, IL-13, IL-15, IL-16, IL-17A, IL-17A/F, IL-17B, IL-17C, IL-21, IL-22, IL-23, IL-27, IL-31, TNF-α, TNF-β, IFN-γ, IP-10, MIP-1α, MIP-1β, MIP-3α, MCP-1, MCP-4, Eotaxin, Eotaxin-3, TARC, MDC, TSLP, CRP, SAA, VEGF-A, VEGF-C, VEGF-D, sTie-2, Flt-1, sICAM-1, sVCAM-1, bFGF, PIGF and GM-CSF. Pearson correlations were calculated using Graph Pad Prism and statistical significance estimated using two tailed tests. Type I error rates were controlled by correcting for multiple comparisons using the Bonferroni method.

### Metagenomic analysis

We included eight previously published shotgun metagenomic studies (Supplementary Table S1)^33,34,57–62^ to investigate the association between relative abundance of genes associated with tryptophan pathways and COVID status. Patients were classified as either COVID or Control and by severity of COVID. Severity status was based on severity at peak when included in the metadata. Mild and moderate severities were grouped together as were severe and critical. Three cohorts were excluded from the severity analysis due to lack of severity data (PRJNA689961, PRJNA714459) or an unbalanced number of severity cases (PRJNA740067). Analyses were conducted on relative abundance data stemming from pathway abundance and gene family data. We utilized HUMAnN3 software to extract Enzyme Commission (EC) numbers from metagenomic sequencing data.^63^ HUMAnN3 facilitates the functional profiling of microbial communities by mapping sequence reads to known enzymatic functions represented by EC numbers. Subsequently, we accessed EC number information from the MetaCyc database using its class hierarchy navigation system.^37^ This system enabled us to retrieve data based on specific categories of interest, thereby enhancing our analysis of the functional diversity within the microbial communities under investigation. Gene abundance in the metagenomic samples was analyzed using the HUMAnN3 pipeline, which initially calculates gene family abundance as Reads Per Kilobase (RPK). Each RPK value is derived by summing alignment scores for all reads mapped to a gene family and then dividing these scores by the length of the reference gene in kilobases for normalization. Additionally, the scores are adjusted to account for cases where a single sequence aligns with multiple reference genes, ensuring accurate representation of gene family abundance. To derive relative abundance (RELAB) from RPK values, the HUMAnN3 renorm_table tool was utilized with the units relab option. This normalization method expresses gene abundance as a proportion of the total gene families within the sample. All statistical analyses were carried out in R 4.3.2. R packages dplyr and ggplot2 were used to transform data and generate graphs.^64,65^ Genes that were present in less than 10% of the samples were excluded from the analysis. Differences between groups were calculated using the Kruskal–Wallis test and Dunn’s multiple comparison test.

### In vitro culture reagents

LPS from *Escherichia coli* was used as a ligand for TLR4, 2‘3’-cGAMP as a ligand for STING, and Poly I:C (LMW)/LyoVec™ as a ligand for RIG-I/MDA-5. These ligands and the reporter system detection solutions (QUANTI-Blue™ and QUANTI-Luc™ 4 Lucia/Gaussia) were all obtained from InvivoGen. Cell culture medium was purchased from Gibco. I3S, ILA, IPA, and IAA were obtained from Sigma-Aldrich. Naïve CD4+ T cell Isolation Kit and human T cell TranAct™ was obtained from Miltenyi Biotec. Cell culture plates were all purchased from Corning. The secretion of IL-10, TNF-α, IP-10, IFN-γ, IFN-α, IFN-β, IL-17A, and IL-22 was measured by ELISA and performed according to manufacturers’ instructions (R&D Systems). Student’s t-test (two tailed) or one-way ANOVA with Tukey’s posttest was performed by GraphPad Prism software version 10.2 to estimate significance differences in cytokine secretion. A p-value less than 0.05 was considered statistically significant. Results were normalized for each human donor to the positive control cytokine values for that donor.

### THP-1 cells

The dual reporter THP-1 monocyte cells were cultured in accordance with InvivoGen’s protocols under standard conditions of 37°C and 5% CO_2_. Dual THP-1 cells allow the simultaneous study of the Nuclear factor kappa B (NF-κB) pathway, by monitoring the activity of secretory embryonic alkaline phosphatase (SEAP), and the IRF3 pathway, by assessing the activity of a secreted luciferase, Lucia luciferase. THP-1 cells were cultured in RPMI 1640 supplemented with 10% (v/v) fetal bovine serum (FBS), 2 mm L-glutamine, 1% (w/v) penicillin/streptomycin, and 100 μg/mL Normocin^TM^. THP-1 cells were pretreated with 10–1000 μM of indoles (I3S/ILA/IPA/IAA) for 30 min, and then stimulated with 3 μg/mL 2′,3′-cGAMP or 1 μg/mL Poly I:C (LMW) (LyoVec™). Negative controls did not receive any stimulation, while the positive control received 3 μg/mL 2′,3′-cGAMP or 1 μg/mL Poly I:C (LMW) (LyoVec™). After 24 h, cell supernatants were transferred into transparent 96-well plates. OD at 620 nm quantified the change in Quanti-Blue color due to SEAP activity, while white 96-well plate was used for luciferase detection with QuantiLuc as the substrate.

### Primary human peripheral blood mononuclear cells (PBMCs)

PBMCs were isolated from blood samples following density gradient centrifugation. Briefly, buffy coat samples were collected from healthy donor volunteers under Irish Blood Transfusion Service approved protocol. The sample was mixed in a 1:2 ratio with 2% FBS in PBS at room temperature in a Ficoll-Paque 1.077 g/mL pre-filled tube (Greiner bio-one). Following centrifugation at 800 g (RCF) without brake for 20 min at room temperature. The mononuclear layer was collected and washed twice, and then centrifuged at 300 g for 8 min at room temperature. After washing, cell pellets were resuspended in complete RPMI medium supplemented with 10% (v/v) heat inactivated FBS, 2 mm L-glutamine, 1% (w/v) penicillin/streptomycin. PBMCs were seeded in 96-well plates at 1 × 10^6^ cells/mL (final volume of 200 μL per well). Before stimulation, cells were pre-incubated for 30 min in the presence or absence of indoles (I3S/ILA/IPA/IAA) at 10–1000 μM. Then, cells were stimulated for 24 h with LPS, or Poly I:C (LMW) (LyoVec™). The negative control contained only culture medium and did not receive any stimulation (Medium–Medium), while the positive control received 100 ng/mL LPS, or 1 μg/mL Poly I:C (LMW) (LyoVec™). Once the incubation time had elapsed, all cultures were centrifuged at 300 g for 8 min at 4°C. The supernatants were harvested and stored at −20°C for the subsequent quantification of pro-inflammatory and anti-inflammatory cytokine secretion.

### Naïve CD4^+^ lymphocyte preparation and stimulation

Untouched Naïve CD4+ T cells were negatively selected from PBMCs with Naïve CD4+ T cell Isolation Kit. Sort purities were routinely above 90% by flow cytometric analysis. PBMC and sorted Naïve CD4+ lymphocytes were seeded in 96-well cell culture plates, and pre-activated with T Cell TransAct™ which is ready-to-use for *in vitro* activation and expansion of human T cells via anti-CD3 and anti-CD28 stimulation for 24 h. Following activation, we added 10–1000 μM of indoles (I3S/ILA/IPA/IAA) into lymphocyte cultures for an additional 48 h. Thereafter, cell supernatants were harvested and stored at −20°C for the subsequent quantification of T cell related cytokine secretion.

### CCK-8 cell viability assay

PBMC or reporter cells were first counted, and approximately 1 × 10^5^ cells per well were seeded in a 96-well cell culture plate. Then, after incubation at 37°C in a humidified atmosphere with 5% CO_2_ overnight, the culture medium was replaced by a series of concentrations of indoles diluted with the corresponding culture medium. Five replicates were made for each measurement, and the maximum time of co-incubation was 48 h. Finally, 20 μL of the CCK-8 reagent was added into each well according to the manufacturer’s instructions, and OD at 450 nm was measured using BioTek microplate reader after incubation for 1–4 h at 37°C.

## Supplementary Material

Supplemental Material

## Data and materials availability

Metagenomic sequencing data is previously published and publicly available as Bioprojects PRJNA740067, PRJNA624223, OEPOO2590, PRJEB43555, PRJNA650244, PRJNA714459, PRJNA689961 and PRJNA660883. Metabolite data is publicly available on FigShare (DOI 10.6084/m9.figshare.18046400).
