# Supplementary material for: Dysrupted microbial tryptophan metabolism associates with SARS-CoV-2 acute inflammatory responses and long COVID
Source: Gut Microbes. 2024 Nov 17;16(1):2429754. doi: 10.1080/19490976.2024.2429754 (PMC11581176; doi:10.1080/19490976.2024.2429754)
Supplement: Supplemental Material [file KGMI_A_2429754_SM1235.zip › Supplementary Table S4.docx]

Table S4. Tryptophan Gene Metadata Correlations

|  | Age | BMI | Male:Female | Antibiotics |
| --- | --- | --- | --- | --- |
| **2.4.2.18** | 0.33 | 0.98 | 0.99 | 0.78 |
| **4.1.1.48** | 0.92 | 0.95 | 0.99 | 0.93 |
| **4.1.3.27** | 0.99 | 0.98 | 0.62 | 0.87 |
| **4.2.1.20** | 0.98 | 0.97 | 0.99 | 0.51 |
| **5.3.1.24** | 0.37 | 0.99 | 0.99 | 0.57 |
| **1.1.1.2** | 0.98 | 0.99 | 0.99 | 0.65 |
| **1.1.1.21** | 0.99 | 0.99 | **0.004** | 0.18 |
| **1.17.1.4** | 0.98 | 0.99 | 0.99 | 0.96 |
| **1.2.1.3** | 0.92 | 0.98 | 0.69 | 0.29 |
| **1.2.1.5** | 0.68 | 0.99 | 0.98 | 0.92 |
| **1.2.1.88** | 0.64 | 0.99 | 0.14 | 0.52 |
| **1.2.3.1** | 0.70 | 0.99 | 0.75 | 0.19 |
| **1.2.7.5** | 0.11 | 0.99 | 0.62 | 0.06 |
| **1.3.1.31** | 0.98 | 0.98 | 0.99 | 0.82 |
| **1.4.3.21** | 0.97 | 0.99 | 0.99 | 0.32 |
| **2.6.1.1** | 0.99 | 0.99 | 0.99 | 0.64 |
| **3.5.1.4** | 0.98 | 0.98 | 0.98 | 0.08 |
| **4.1.1.74** | 0.88 | 0.98 | 0.99 | 0.15 |
| **4.1.99.1** | 0.70 | 0.99 | 0.99 | 0.09 |

Adjusted p-values are illustrated for each comparison.
