# Supplementary material for: Dysrupted microbial tryptophan metabolism associates with SARS-CoV-2 acute inflammatory responses and long COVID
Source: Gut Microbes. 2024 Nov 17;16(1):2429754. doi: 10.1080/19490976.2024.2429754 (PMC11581176; doi:10.1080/19490976.2024.2429754)
Supplement: Supplemental Material [file KGMI_A_2429754_SM1235.zip › Supplementary Table S1.docx]

Table S1. Metagenomic studies.

| **BioProject** | **Reference** | **Sequencing type** | **Total** | **Controls** | **Mild/Moderate COVID19** | **Severe/Fatal COVID19** | **Uncategorized COVID19 Severity** |
| --- | --- | --- | --- | --- | --- | --- | --- |
| PRJNA740067 | Zhou T, Wu J, Zeng Y, Li J, Yan J, Meng W *et al* (2022). SARS-CoV-2 triggered oxidative stress and abnormal energy metabolism in gut microbiota. *MedComm* **3:** e112. | Shotgun metagenome | 26 | 13 | 12 | 1 |  |
| PRJNA624223 | Zuo T, Zhang F, Lui GCY, Yeoh YK, Li AYL, Zhan H *et al* (2020). Alterations in Gut Microbiota of Patients With COVID-19 During Time of Hospitalization. *Gastroenterology* **159:** 944-955.e948. | Shotgun metagenome | 30 | 15 | 10 | 5 |  |
| OEP002590 | Sun Z, Song Z-G, Liu C, Tan S, Lin S, Zhu J *et al* (2022). Gut microbiome alterations and gut barrier dysfunction are associated with host immune homeostasis in COVID-19 patients. *BMC Medicine* **20:** 24. | Shotgun metagenome | 71 | 8 | 50 | 13 |  |
| PRJEB43555 | Li S, Yang S, Zhou Y, Disoma C, Dong Z, Du A *et al* (2021). Microbiome Profiling Using Shotgun Metagenomic Sequencing Identified Unique Microorganisms in COVID-19 Patients With Altered Gut Microbiota. *Frontiers in Microbiology* **12**. | Shotgun metagenome | 65 | 19 | 36 | 10 |  |
| PRJNA650244 | Yeoh YK, Zuo T, Lui GC-Y, Zhang F, Liu Q, Li AYL *et al* (2021). Gut microbiota composition reflects disease severity and dysfunctional immune responses in patients with COVID-19. *Gut* **70:** 698. | Shotgun metagenome | 178 | 78 | 92 | 8 |  |
| PRJNA714459 | Liu Q, Mak JWY, Su Q, Yeoh YK, Lui GC-Y, Ng SSS *et al* (2022). Gut microbiota dynamics in a prospective cohort of patients with post-acute COVID-19 syndrome. *Gut***:** gutjnl-2021-325989. | Shotgun metagenome | 115 | 67 |  |  | 48 |
| PRJNA689961 | Zhang F, Wan Y, Zuo T, Yeoh YK, Liu Q, Zhang L *et al* (2022). Prolonged Impairment of Short-Chain Fatty Acid and L-Isoleucine Biosynthesis in Gut Microbiome in Patients With COVID-19. *Gastroenterology* **162:** 548-561.e544. | Shotgun metagenome | 136 | 70 |  |  | 66 |
| PRJNA660883 | Britton GJ, Chen-Liaw A, Cossarini F, Livanos AE, Spindler MP, Plitt T *et al* (2021). Limited intestinal inflammation despite diarrhea, fecal viral RNA and SARS-CoV-2-specific IgA in patients with acute COVID-19. *Scientific Reports* **11:** 13308. | Shotgun metagenome | 29 | 0 | 17 | 12 |  |
