# Supplementary material for: Dysrupted microbial tryptophan metabolism associates with SARS-CoV-2 acute inflammatory responses and long COVID
Source: Gut Microbes. 2024 Nov 17;16(1):2429754. doi: 10.1080/19490976.2024.2429754 (PMC11581176; doi:10.1080/19490976.2024.2429754)
Supplement: Supplemental Material [file KGMI_A_2429754_SM1235.zip › Supplementary_Figures.docx]

✱✱✱✱

✱✱✱✱


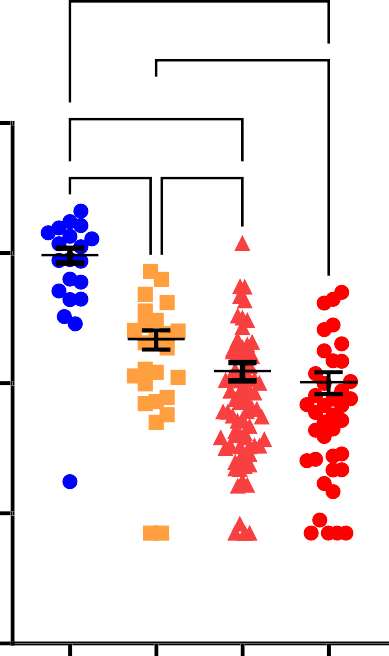


✱

✱✱✱✱

✱✱ ✱

✱✱✱✱


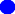

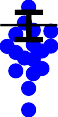
**100 10 10**


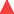


✱

✱✱✱✱


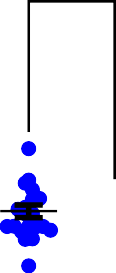


✱✱

✱✱✱✱

✱

**10**

**1 1 1**

**Indolepropionate**

**Indoleacetate**

**Indolelactate**

**0.1**

**0.01**

**0.1**

**0.1**

*Figure S1. Serum microbial metabolites in hospitalised COVID-19 patients.*

Serum levels of microbial tryptophan derived metabolites are illustrated. Results are expressed as mean and standard deviation of the scaled medians for each metabolite. Differences between groups are calculated using the Kruskal-Wallis test and Dunn’s multiple comparison test (*p<0.05,

**p<0.01, ****p<0.0001).

✱✱✱✱

✱✱✱✱


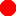

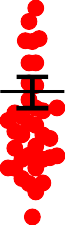

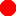

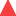

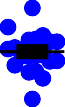


✱✱✱

✱✱

**10 10**


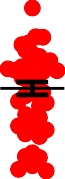

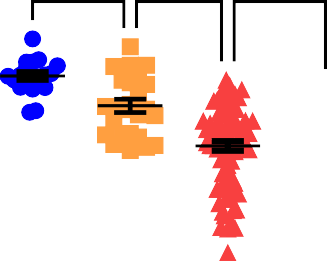


✱✱✱✱

✱✱✱✱

✱✱✱ ✱✱✱✱ ✱

**1 1**

**Tryptophan**

**C-glycosyltryptophan**

**0.1**

**0.1**

*Figure S2. Serum metabolites in hospitalised COVID-19 patients.*

Serum levels of tryptophan and the host modified derivative C-glycosyltryptophan are illustrated. Results are expressed as mean and standard deviation of the scaled medians for each metabolite. Differences between groups are calculated using the Kruskal-Wallis test and Dunn’s multiple comparison test (*p<0.05, **p<0.01, ***p<0.001, ****p<0.0001).

✱✱


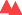

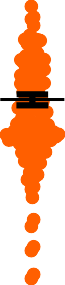

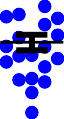


✱✱✱✱

✱✱✱

✱✱✱✱

**10 10**


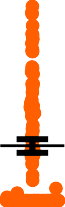


✱✱✱✱

✱✱✱✱

**1**

**5-hydroxyindole sulfate**

**3-indoxyl sulfate**

**1**

**0.1**

**0.1**

**Control COVID Survive Fatal**

**0.01**

**Control COVID Survive Fatal**

*Figure S3. Serum microbial metabolites in hospitalised COVID-19 patients.*

Serum levels of host modified bacterial indoles are illustrated. Results are expressed as mean and standard deviation of the scaled medians for each metabolite. Differences between groups are calculated using the Kruskal-Wallis test and Dunn’s multiple comparison test (*p<0.05, **p<0.01,

***p<0.001, ****p<0.0001).

**2.5 25**


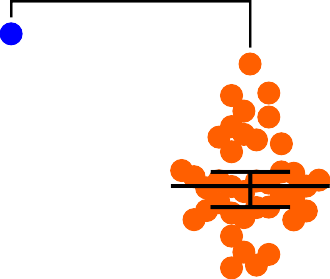

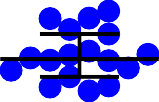


✱✱

✱✱

**2.0 20**


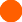

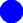


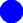


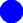


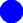
**1.5 15**


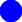

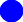

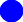

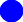

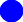


**Tryptophan**

**Indolepropionate**

**1.0 10**


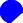


**0.5 5**

**0.0**

**1.5**


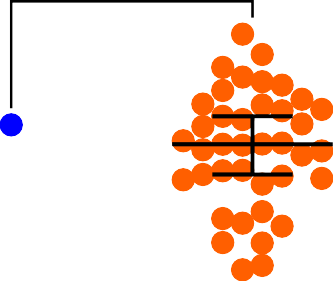

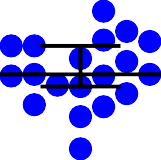


✱✱✱✱

**0**

**CONTROLS Long COVID**

**10**


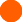

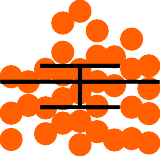

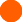

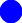

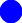

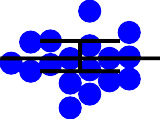

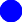


✱

**8**

**1.0**

**C-glycosyltryptophan**

**indoleacetate**

**6**

**4**

**0.5**

**2**

**0.0**

**CONTROLS Long COVID**

**0**

**CONTROLS Long COVID**

*Figure S4. Long COVID serum metabolites.*

Serum levels of tryptophan, C-glycosyltryptophan, indolepropionate and indoleacetate are illustrated. Results are expressed as mean and standard deviation of the scaled medians for each metabolite. Differences between groups are calculated using the Mann Whitney test (*p<0.05,

**p<0.01, ****p<0.0001).

**10000**

✱✱✱

**100**

✱✱✱

**100**

✱✱✱✱

**10000**

✱✱✱✱

**10000**


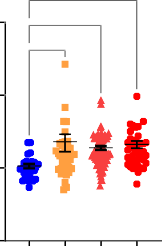


✱✱✱✱

✱✱

✱✱✱✱

**1000**


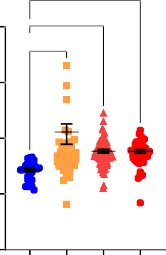


✱✱✱✱

✱✱✱✱

✱✱✱✱

**1000**


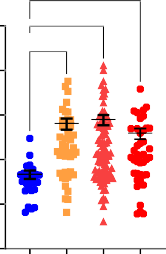


✱✱✱✱

✱✱✱✱

**100**

**IFN-**γ **[pg/ml]**

**10**

**1**

**0.1**

**10**

**1**


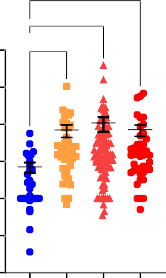


✱✱✱✱

✱✱✱✱

**IL-1**β **[pg/ml]**

**0.1**

**0.01**

**0.001**

**0.0001**

**10**

**1**


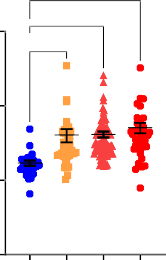


✱✱✱✱

✱✱✱✱

**TNF-**α **[pg/ml]**

**0.1**

**1000**

**100**

**MIP-1β [pg/ml]**

**10**

**1000**

**100**

**MIP-1α [pg/ml]**

**10**

**1**

**100**

**10**


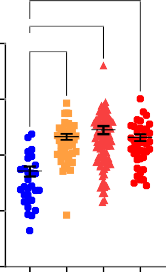


✱✱✱✱

✱✱✱✱

**bFGF [pg/ml]**

**1**

**0.1**

✱✱✱✱

✱✱

✱✱✱✱


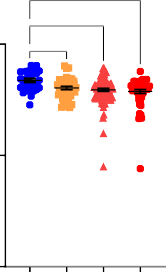


✱✱✱

✱

✱✱✱✱

✱✱✱✱

✱✱✱ ✱✱✱✱

✱✱✱✱

✱✱✱✱

✱✱✱✱


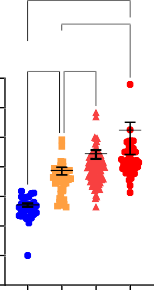


✱✱✱✱

✱✱✱✱

✱✱✱✱ ✱✱

**1000**

**1000**

**1000**

**1000**


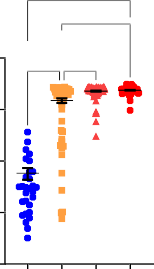


✱✱✱

✱✱✱✱

✱✱✱✱ ✱✱✱

**10000**

**1000**

✱✱✱

✱✱✱✱

✱✱✱✱ ✱

**100**

**10**

**IL-13 [pg/ml]**

**100**

**100**

**SAA [mg/L]**

**1000**

**100**

**100**

**100**


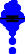


✱✱

✱✱✱

**sTie-2 [pg/ml]**

**CRP [mg/L]**

**1 10**

**10**

**10**

**10**

**IL-6 [pg/ml]**

**IL-10 [pg/ml]**

**1**

**0.1**

**0.01**

**1 1**

**0.1**

**1**

**0.01**

**10 0.**

**0.**

**0.1**

**0.001**

**100000**

✱✱✱✱

**1000**

✱✱✱✱

**1000**


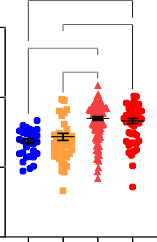


✱✱✱

✱✱✱✱

✱✱✱✱

✱✱

**100**

✱✱✱✱

✱✱


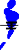


✱

**100**

**10000**

✱✱✱✱

**100 10 10**


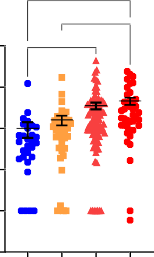


✱✱

✱✱✱


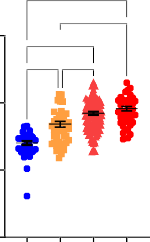


✱✱✱✱

✱✱✱✱

✱✱ ✱✱


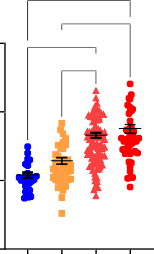


✱✱✱✱

✱✱✱✱

✱✱✱✱


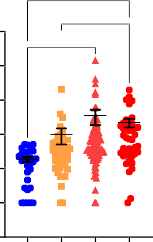


✱

✱✱✱✱

**10000**

**IL-27 [pg/ml]**

**10 100**

**1000**

**1 1**

**IL-22 [pg/ml]**

**IL-4 [pg/ml]**

**IL-16 [pg/ml]**

**1000**

**100**

**1**

**0.1**

**IL-2 [pg/ml]**

**0.01**

**0.001**

**10**

**IL-7 [pg/ml]**

**1**

**0.1**

**0.01**

**0.001**

**0.1**

**0.01**

**0.0**

**100**

**10**

**10000**


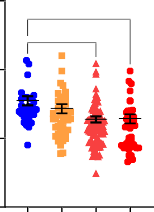


✱✱✱✱

✱✱✱✱

**10000**

✱✱✱✱

**10000**


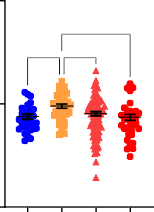


✱✱

✱ ✱

**100**

✱✱✱✱

**100000**


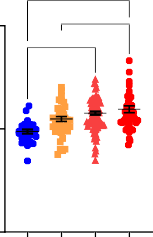


✱

✱✱✱

✱✱✱✱

**107**


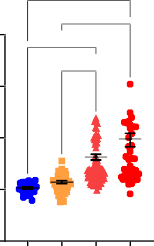


✱✱✱✱

✱✱✱✱

✱✱✱✱

✱✱✱✱

**10000**


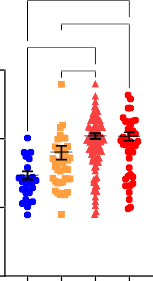


✱

✱✱✱✱

✱✱✱


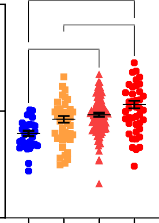


✱

✱✱✱✱

**VEGF-D [pg/ml]**

**sVCAM-1 [pg/ml]**

**1000**

**TARC [pg/ml]**

**1000**

**VEGF-A [pg/ml]**

**1000**

**10 1000**

**PIGF [pg/ml]**

**sFlt1 [pg/ml]**

**106**

**100**

**100**

**100**

**10**

**100**

**10**

**1 10**

**105**

✱ ✱✱✱✱


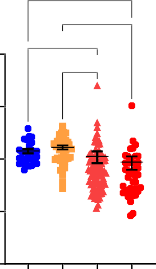


✱✱✱✱

✱✱✱

✱✱✱✱

✱✱✱✱

✱✱✱✱

**10000**

**1000**

**VEGF [pg/ml]**

✱✱✱✱

**100**

**10**

**IL-17A [pg/ml]**

**10000**

**1000**


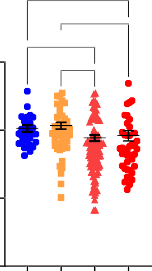


✱

✱✱

✱✱

**IL-12/IL-23p40 [pg/ml]**

**100**

**1000**

**100**


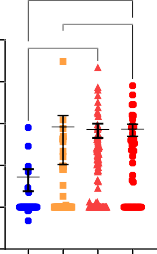


✱

✱✱✱

**10**

**IL-17C [pg/ml]**

**1000**

**100**


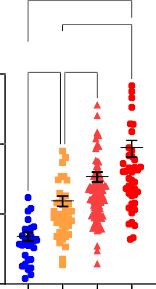


✱✱✱✱

✱✱✱✱

✱✱ ✱✱

**MIP-3**α **[pg/ml]**

**100**


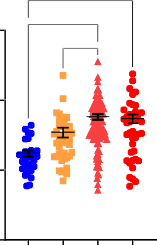


✱✱✱✱

✱✱

**1**

**10**

**1**

**10**

**0.1**

**0.1**

**1**

**0.01 1**

**10**

*Figure S5. Serum cytokine levels.*

Results are expressed as mean and standard deviation. Differences between groups are calculated using the Kruskal-Wallis test and Dunn’s multiple comparison test (*p<0.05, **p<0.01, ***p<0.001,

****p<0.0001).


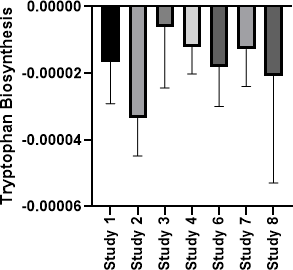


*Figure S6. Tryptophan biosynthesis in COVID-19 patients compared to healthy controls.*

Each COVID-19 study that included healthy volunteers was separately analysed. The mean pathway relative abundance for healthy controls in each study was subtracted from individual COVID-19 patient tryptophan pathway relative abundance in that specific study. Results are expressed as mean difference and standard deviation for each study.


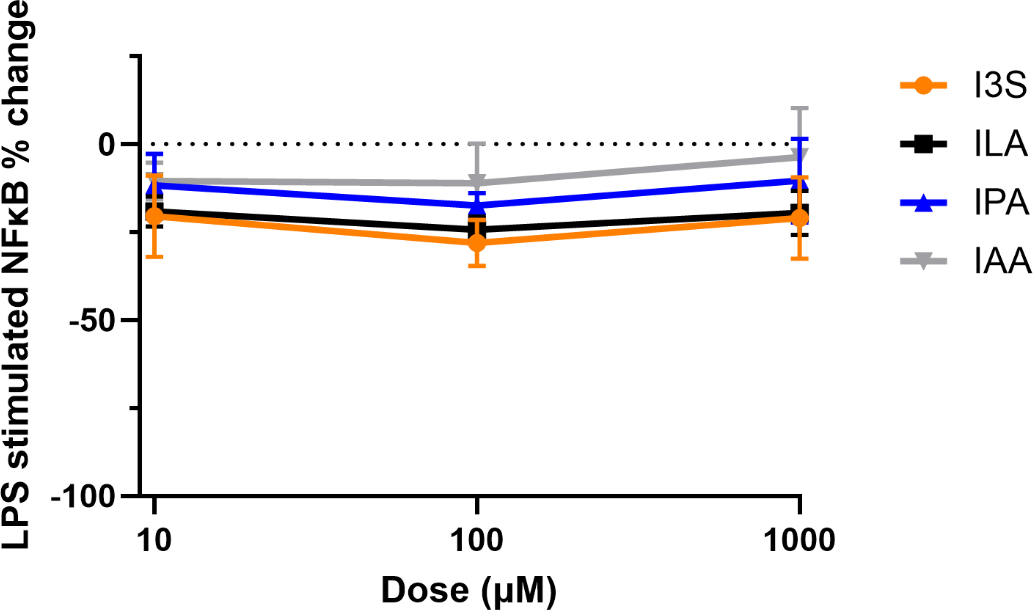


*Figure S7. LPS-induced NF-*κ*B activation in THP-1 cells.*

Co-incubation with microbial tryptophan metabolites did not significantly influence LPS-induced NF-κB activation. IPA – Indole-3-propionate; ILA – Indole-3-lactate; IAA – Indole-3-acetate; I3S 3- indoxyl sulfate.
