# Supplementary material for: Dysrupted microbial tryptophan metabolism associates with SARS-CoV-2 acute inflammatory responses and long COVID
Source: Gut Microbes. 2024 Nov 17;16(1):2429754. doi: 10.1080/19490976.2024.2429754 (PMC11581176; doi:10.1080/19490976.2024.2429754)
Supplement: Supplemental Material [file KGMI_A_2429754_SM1235.zip › Supplementary Table S3.docx]

Table S3. Tryptophan Gene Presence in Human Faecal Samples

|  |  |  | COVID Severity | | |
| --- | --- | --- | --- | --- | --- |
|  | Control n=270 | COVID n=380 | Mild/Moderate n=217 | Severe/Fatal n=49 | Uncategorized n=114 |
| **2.4.2.18** | 100% | 99.7% | 99.5% | 100% | 100% |
| **4.1.1.48** | 100% | 99.7% | 99.5% | 100% | 100% |
| **4.1.3.27** | 100% | 100% | 100% | 100% | 100% |
| **4.2.1.20** | 100% | 99.7% | 99.5% | 100% | 100% |
| **5.3.1.24** | 100% | 99.7% | 99.5% | 100% | 100% |
| **1.1.1.2** | 99.6% | 97.6% | 97.2% | 98.0% | 100% |
| **1.1.1.21** | 97% | 79.7% | 79.3% | 69.4% | 88.6% |
| **1.17.1.4** | 99.2% | 97.1% | 95.9% | 100% | 98.2% |
| **1.2.1.3** | 84.4% | 78.2% | 79.3% | 79.6% | 76.3% |
| **1.2.1.5** | 76.3% | 66.1% | 65.4% | 65.3% | 67.5% |
| **1.2.1.88** | 98.1% | 90.8% | 90.7% | 83.6% | 93.8% |
| **1.2.3.1** | 60.3% | 34.7% | 35.0% | 14.3% | 42.9% |
| **1.2.7.5** | 82.5% | 60.3% | 55.3% | 49.0% | 74.5% |
| **1.3.1.31** | 58.9% | 62.6% | 58.8% | 63.2% | 69.3% |
| **1.4.3.21** | 59.6% | 40.0% | 38.7% | 36.7% | 43.9% |
| **2.6.1.1** | 100% | 99.7% | 99.5% | 100% | 100% |
| **3.5.1.4** | 98.9% | 87.9% | 84.8% | 79.6% | 97.3% |
| **4.1.1.74** | 25.6% | 23.9% | 20.3% | 30.6% | 28.1% |
| **4.1.99.1** | 95.9% | 90.3% | 90.3% | 85.7% | 92.1% |
