# Supplementary figures and images for: Dysrupted microbial tryptophan metabolism associates with SARS-CoV-2 acute inflammatory responses and long COVID
Source: Gut Microbes. 2024 Nov 17;16(1):2429754. doi: 10.1080/19490976.2024.2429754 (PMC11581176; doi:10.1080/19490976.2024.2429754)

Table S2. Tryptophan metabolism genes.
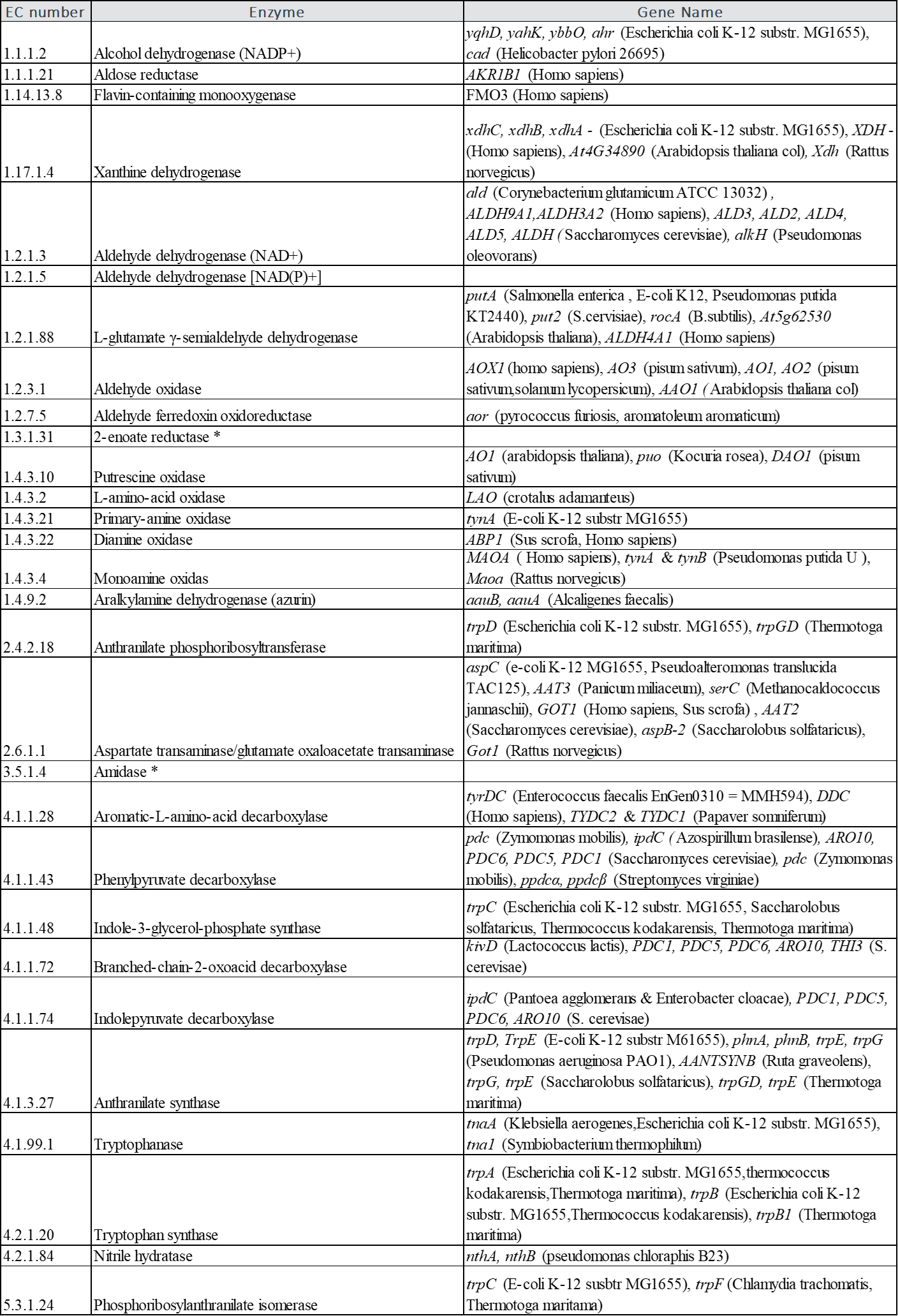

Supplement: Supplemental Material [file KGMI_A_2429754_SM1235.zip › Supplementary Table S2.docx]
